# Randomized Clinical Trial: Efficacy of a New Synbiotic in Adults with Metabolic Syndrome

**DOI:** 10.5195/cajgh.2013.111

**Published:** 2014-01-24

**Authors:** Almagul Kushugulova, Valerii Benberin, Raushan Karabayeva, Saule Saduakhasova, Samat Kozhakhmetov, Gulnara Shakhabayeva, Indira Tynybayeva, Talgat Nurgozhin, Zhaxybay Zhumadilov

**Affiliations:** 1 Center for Life Sciences, Nazarbaev University, Astana, Kazakhstan; 2 Medical Center of President’s Affairs Administration of Republic of Kazakhstan, Astana, Kazakhstan

**Keywords:** synbiotics, efficacy, metabolic syndrome

## Abstract

**Introduction:**

Metabolic syndrome is a lifestyle disease and is a frequent problem among the adult population. Human gut microbiota plays a key role in the development of metabolic syndrome. Recently, the gut microbiota has emerged as an important contributor to the development of obesity and metabolic disorders through its interactions with environmental (e.g. diet) and genetic factors. The aim of this study was to research the effects of synbiotic on the gut microbiota and host metabolism.

**Methods:**

We conducted a double-blind, randomized, placebo-controlled trial. Our sample included 180 adults (ages 30–89) with symptoms of metabolic syndrome, who were allocated to either placebo or synbiotic group. The main inclusion criteria were: blood pressure of around 130/90 mmHg; raised fasting plasma glucose (FPG) >100 mg/dL (5.6 mmol/L), previous diagnosis of type 2 diabetes, dyslipidemia triglycerides (TG) of 1.70 mmol/L, a high-density lipoprotein cholesterol (HDL-C) of 0.90 mmol/L in males and 1.0 mmol/L in females, and central obesity with a waist/hip ratio > 0.90 in males or > 0.85 in females or a body mass index > 30 kg/m^2^.

**Results:**

We enrolled 90 adults in the placebo group and 90 in the synbiotic group. The two groups had similar demographic and clinical characteristics. Consent was signed by all patients. All patients underwent clinical and laboratory evaluation, including complete blood tests, glucose test, glycosylated hemoglobin, total cholesterol and triglycerides, cholesterol, LDL, HDL plasma, immunogram, and coprogram. All patients were interviewed with a questionnaire that included 200 questions related to diet, lifestyle, and health. Synbiotic were used by patients in a dose of 200 grams twice a day. The duration of applying of the synbiotic was 90 days.

To study the composition of the intestinal microbiota, stool samples were collected before and after applying the synbiotic. The microbial composition will be determined by analyzing the locus of 16S rDNA.

**Conclusion:**

This ongoing study is currently undergoing microbial composition analysis in order to establish the efficacy of the new synbiotic in adults with metabolic syndrome.

